# From Molecular Precision to Clinical Practice: A Comprehensive Review of Bispecific and Trispecific Antibodies in Hematologic Malignancies

**DOI:** 10.3390/ijms26115319

**Published:** 2025-06-01

**Authors:** Behzad Amoozgar, Ayrton Bangolo, Maryam Habibi, Christina Cho, Andre Goy

**Affiliations:** 1 Department of Hematology and Oncology, John Theurer Cancer Center, Hackensack University Medical Center, Hackensack, NJ 07601, USA; 2 Tulane National Primate Research Center, Tulane University, New Orleans, LA 70118, USA; 3 Division of Stem Cell Transplant and Cellular Therapy, John Theurer Cancer Center, Hackensack University Medical Center, Hackensack, NJ 07601, USA; 4 Division of Lymphoma, John Theurer Cancer Center, Hackensack University Medical Center, Hackensack, NJ 07601, USA

**Keywords:** bispecific antibodies, trispecific antibodies, hematologic malignancies, immunotherapy, T-cell engagers

## Abstract

Multispecific antibodies have redefined the immunotherapeutic landscape in hematologic malignancies. Bispecific antibodies (BsAbs), which redirect cytotoxic T cells toward malignant targets via dual antigen engagement, are now established components of treatment for diseases such as acute lymphoblastic leukemia (ALL), diffuse large B-cell lymphoma (DLBCL), follicular lymphoma (FL), and multiple myeloma (MM). Clinical trials of agents like blinatumomab, glofitamab, mosunetuzumab, and teclistamab have demonstrated deep and durable responses in heavily pretreated populations. Trispecific antibodies (TsAbs), although still investigational, represent the next generation of immune redirection therapies, incorporating additional tumor antigens or co-stimulatory domains (e.g., CD28, 4-1BB) to mitigate antigen escape and enhance T-cell persistence. This review provides a comprehensive evaluation of BsAbs and TsAbs across hematologic malignancies, detailing molecular designs, mechanisms of action, therapeutic indications, resistance pathways, and toxicity profiles including cytokine release syndrome (CRS), immune effector cell-associated neurotoxicity syndrome (ICANS), cytopenias, and infections. We further discuss strategies to mitigate adverse effects and resistance, such as antigen switching, checkpoint blockade combinations, CELMoDs, and construct optimization. Notably, emerging platforms such as tetrafunctional constructs, checkpoint-integrated multispecifics, and protease-cleavable masking designs are expanding the therapeutic index of these agents. Early clinical evidence also supports the feasibility of applying multispecific antibodies to solid tumors. Finally, we highlight the transformative role of artificial intelligence (AI) and machine learning (ML) in multispecific antibody development, including antigen discovery, biomarker-driven treatment selection, toxicity prediction, and therapeutic optimization. Together, BsAbs and TsAbs illustrate the convergence of molecular precision, clinical innovation, and AI-driven personalization, establishing a new paradigm for immune-based therapy across hematologic and potentially solid tumor malignancies.

## 1. Introduction

Bispecific (BsAbs) and trispecific antibodies (TsAbs) represent transformative innovations in the immunotherapeutic landscape of hematologic malignancies. By simultaneously engaging tumor-associated antigens and redirecting immune effector cells—most often T cells—these engineered molecules enable potent immune activation, independent of major histocompatibility complex (MHC) restriction. BsAbs have already reshaped treatment paradigms in diseases such as B-cell non-Hodgkin lymphoma, multiple myeloma, and acute lymphoblastic leukemia, offering new therapeutic strategies, particularly in relapsed or refractory settings [1,2,3].

BsAbs, including CD3-engaging constructs like bispecific T-cell engagers (BiTEs) and Fc-containing IgG-like formats, are now approved by the FDA and EMA across several indications. Agents such as blinatumomab (CD19 × CD3) and teclistamab (BCMA × CD3) have demonstrated high response rates and manageable toxicity profiles in heavily pretreated populations [4,5,6,7]. The development of fixed-duration regimens, subcutaneous administration, and combination strategies with immunomodulatory agents (IMiDs) or anti-CD38 monoclonal antibodies continues to expand the clinical utility and accessibility of BsAb-based therapies.

TsAbs remain investigational but offer the promise of further addressing resistance mechanisms such as antigen escape, T-cell exhaustion, and tumor heterogeneity. By incorporating additional tumor-associated antigens or co-stimulatory domains (e.g., CD28, 4-1BB), TsAbs aim to enhance T-cell activation, broaden antigen coverage, and prolong immune responses. Early-phase clinical studies of constructs such as CD38/CD28 × CD3 in multiple myeloma and CD19/CD22/CD3 formats in B-cell malignancies have provided compelling proof-of-concept data [8,9,10].

In this review, we comprehensively evaluate the molecular design, mechanistic underpinnings, clinical applications, and resistance patterns of bispecific and trispecific antibodies in hematologic malignancies. We discuss their therapeutic integration across disease contexts—including B-cell lymphomas, acute leukemias, multiple myeloma, and myeloid neoplasms—alongside an analysis of emerging toxicity profiles, including cytokine release syndrome (CRS), neurotoxicity, cytopenias, and infections. Furthermore, we explore next-generation platforms beyond trispecifics, the expansion of multispecifics into solid tumors, and the transformative role of artificial intelligence (AI) in antibody engineering and clinical deployment. Together, these insights underscore the central and expanding roles of BsAbs and TsAbs in next-generation immune-based oncology.

## 2. Molecular Design of Bispecific and Trispecific Constructs

Multispecific antibodies—including BsAbs and TsAbs—are engineered molecules designed to simultaneously engage multiple targets, enabling the redirection of immune effector cells to malignant cells. In hematologic malignancies, these constructs have demonstrated considerable therapeutic potential, addressing challenges such as antigen escape, tumor heterogeneity, and immune evasion [11,12,13].

BsAbs are typically designed to bind both a tumor-associated antigen (TAA) and an immune effector cell receptor, most commonly CD3 on T cells. This dual engagement facilitates immune synapse formation and targeted cytolysis of malignant cells. Structurally, BsAbs exist in two principal formats: full-length IgG-like antibodies and fragment-based constructs. Full-length BsAbs retain an Fc region that extends half-life via FcRn-mediated recycling and are often engineered with silenced Fc domains to minimize FcγR-mediated toxicities such as ADCC and CDC. Representative agents include glofitamab, mosunetuzumab, and epcoritamab, targeting CD20 × CD3, with demonstrated efficacy in B-cell non-Hodgkin lymphoma [14,15,16]. Their pharmacokinetic advantages allow for intermittent dosing and greater patient convenience [17,18].

Fragment-based constructs, such as bispecific T-cell engagers (BiTEs), are composed of two single-chain variable fragments (scFvs) connected by linkers and lack an Fc domain. These constructs offer high tumor penetration but require continuous infusion due to their short half-lives. Blinatumomab, a CD19 × CD3 BiTE, remains a foundational therapy for relapsed or refractory B-ALL [19]. To address the limitations of conventional BiTEs, newer molecules—such as half-life extended BiTEs (e.g., AMG-701)—have been developed to enable less frequent administration without compromising efficacy [20,21].

Advances in BsAb design have led to innovative configurations that improve antigen avidity. Glofitamab, for instance, uses a 2:1 CD20:CD3 format to enhance targeting of low-density antigens. Similarly, teclistamab (BCMA × CD3) has demonstrated robust activity in triple-class refractory multiple myeloma, highlighting the clinical impact of BsAb-based therapies [22,23,24,25]. In addition, alternative constructs that engage FcγRIIIa (CD16) activate NK cell–mediated cytotoxicity and may benefit patients with T-cell dysfunction [26,27].

TsAbs expand upon BsAb platforms by incorporating tri-specificity—engaging three distinct targets to broaden tumor recognition and strengthen immune activation. Structural formats include IgG-like constructs with Fc regions for stability and scFv-based trispecific T-cell engagers (TriTEs), which offer compact design and enhanced tumor penetration [28,29]. TsAbs may simultaneously target two TAAs and CD3, or combine CD3 with co-stimulatory molecules (e.g., CD28), enhancing T-cell activation, proliferation, and resistance to exhaustion [30,31,32]. Some constructs also recruit CD16 on NK cells, expanding their utility in immune-compromised settings [33,34].

Several TsAbs illustrate these principles. Constructs such as CD19/CD22/CD3 have demonstrated efficacy in B-cell malignancies by addressing antigen escape in heterogeneous populations [35,36]. Others, such as CD3/CD28/TAA or CD20/CD79b/CD3, have shown potential in refractory settings like multiple myeloma and non-Hodgkin lymphoma [37,38]. An investigational CD38/CD28 × CD3 construct (SAR442257) has shown preclinical efficacy in myeloma models, combining dual targeting and T-cell co-stimulation [39,40].

**Figure 1:**
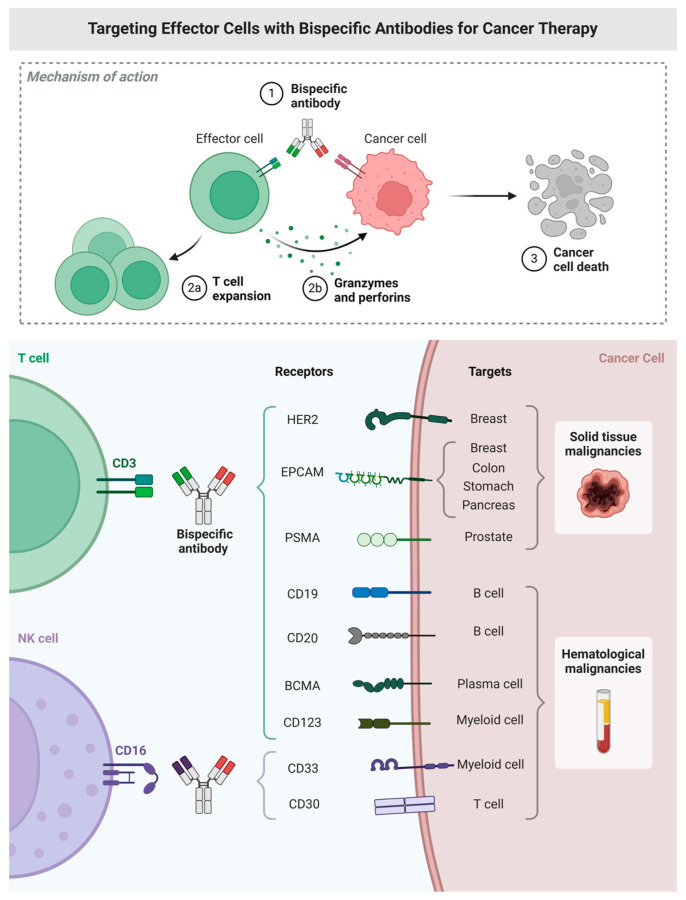
Targeting effector cells with bispecific antibodies for cancer therapy—illustrating mechanisms of T-cell and NK-cell redirection.

Overall, the molecular evolution of BsAbs and TsAbs reflects a strategic response to the immunologic complexities of hematologic cancers. By combining antigen targeting, immune redirection, and costimulatory activation, these multispecific platforms provide flexible and potent tools with the potential to overcome limitations of traditional therapies. Continued refinement in design, pharmacokinetics, and immune modulation is expected to broaden their clinical impact across hematologic malignancies (Figure 1).

## 3. Therapeutic Indications of Bispecific and Trispecific Antibodies in Hematologic Malignancies

Multispecific antibodies—including BsAbs and TsAbs—are reshaping the therapeutic landscape of hematologic malignancies by addressing challenges such as antigen heterogeneity, immune escape, and treatment resistance. Through engagement of tumor-associated antigens (TAAs) and redirection of immune effector cells, these constructs enable potent, targeted immune responses that are independent of MHC restriction and tumor antigen presentation pathways.

BsAbs have already been integrated into clinical practice across multiple hematologic malignancies, with approvals granted for various CD3-engaging constructs. Their modular design allows for tailored pharmacokinetics, antigen flexibility, and combinatorial potential. These attributes have proven especially beneficial in relapsed/refractory settings and in patients with limited treatment options.

TsAbs are a next-generation extension of this platform, incorporating a third functional domain—most often a second TAA or a co-stimulatory molecule (e.g., CD28, CD16). Preclinical and early clinical studies suggest that TsAbs may enhance T-cell persistence, overcome immune escape, and broaden tumor recognition. Additionally, formats that engage innate immunity or dual adaptive checkpoints are under investigation, expanding the potential utility of these agents.

In the sections that follow, we examine the role of BsAbs and TsAbs within individual disease contexts—highlighting clinical trial evidence, resistance patterns, and emerging therapeutic strategies relevant to B-cell malignancies, myeloid neoplasms, and plasma cell dyscrasias.

### 3.1. Acute Lymphoblastic Leukemia (ALL)

Acute lymphoblastic leukemia (ALL) has been a major area of focus for multispecific antibody development due to the consistent expression of tumor-associated antigens such as CD19 and CD22 on B-lineage blasts. Bispecific antibodies (BsAbs), particularly blinatumomab—a CD19 × CD3 bispecific T-cell engager (BiTE)—have significantly advanced the treatment paradigm for relapsed or refractory (R/R) ALL. In the pivotal TOWER trial, blinatumomab demonstrated superior efficacy over standard chemotherapy in adults with R/R Philadelphia chromosome (Ph)-negative ALL, achieving a median overall survival (OS) of 7.7 months versus 4.0 months (hazard ratio [HR] 0.71, 95% confidence interval [CI]: 0.55–0.93) [41,42]. In pediatric populations, blinatumomab also showed a meaningful benefit; among children with high-risk first-relapse ALL, event-free survival (EFS) was 66.2% with blinatumomab versus 27.1% with chemotherapy prior to allogeneic stem cell transplantation (allo-SCT) (HR 0.33, 95% CI: 0.18–0.61) [41,42]. These results support blinatumomab’s role as a bridging therapy to allo-SCT and have led to its incorporation into standard treatment regimens for both adult and pediatric patients.

To address resistance mechanisms such as CD19 antigen loss and to enhance immune engagement, trispecific antibodies (TsAbs) are under development. Preclinical studies of CD19/CD22/CD3 TsAbs have shown that they effectively target heterogeneous leukemic populations and eliminate primary B-ALL cells in xenograft models [43,44]. These constructs aim to mitigate immune escape and broaden tumor targeting by engaging multiple B-cell antigens simultaneously. Early data suggest improved cytotoxicity and durability of response compared to conventional BsAbs.

Further, incorporation of co-stimulatory domains into TsAbs—such as CD28—has been explored in preclinical models to augment T-cell activation and persistence. While constructs combining CD3, CD19 or CD22, and CD28 have demonstrated enhanced efficacy in high-burden ALL models in vitro, no clinical-stage CD28-containing trispecifics for ALL have been developed to date [45,46].

Collectively, these developments highlight the evolving role of both bispecific and trispecific platforms in ALL. BsAbs have already established themselves as effective therapies in the relapsed/refractory setting, while TsAbs offer the potential to overcome resistance and further deepen responses in future applications.

### 3.2. Acute Myeloid Leukemia (AML)

Acute myeloid leukemia (AML) remains a challenging malignancy, particularly in relapsed or refractory settings, due to disease heterogeneity, leukemic stem cell persistence, and an immunosuppressive microenvironment. Among bispecific antibodies (BsAbs), CD33 and CD123 have emerged as the most extensively studied targets.

Flotetuzumab, a CD123 × CD3 DART BsAb, demonstrated an overall response rate (ORR) of 24% in a phase I/II trial of relapsed/refractory AML, with a manageable safety profile, including mostly low-grade cytokine release syndrome (CRS) [47]. XmAb14045, another CD123-targeting BsAb, has shown encouraging activity in early-phase studies, reinforcing CD123 as a promising therapeutic target [48]. Additionally, BsAbs targeting CD33, such as JNJ-67571244, have entered clinical evaluation, with evidence of preliminary anti-leukemic activity [49,50,51,52].

Efforts to target CLL-1, a leukemic stem cell-associated antigen, are ongoing to enhance durability of remission and minimize off-target toxicities [49,53]. These strategies aim to refine BsAb-based approaches in AML, especially in refractory disease, where treatment options are limited.

TsAbs expand on this by incorporating additional targeting or co-stimulatory mechanisms. For instance, a trispecific killer engager (TriKE) targeting CLEC12A/CD16/IL-15 uses innate immune mechanisms to kill AML cells via NK activation and IL-15-driven proliferation [54]. Other constructs such as CD38/CD3 × CD28 (e.g., SAR442257) leverage CD28-mediated T-cell co-stimulation and are under preclinical or early clinical evaluation. Although SAR442257 was primarily developed for multiple myeloma and NHL, preclinical studies suggest potential relevance in AML [55]. Additionally, multi-antigen constructs co-engaging CD33, CD123, and CD3 are being developed to overcome antigenic heterogeneity, with preclinical studies showing potent anti-AML activity in xenograft models [56].

Together, BsAbs and TsAbs represent complementary approaches in AML: BsAbs provide targeted cytotoxicity against key myeloid markers, while TsAbs may overcome immune evasion by engaging additional pathways or cell types. Ongoing trials are expected to further clarify their optimal application and sequencing in the evolving AML treatment landscape.

### 3.3. B-Cell Non-Hodgkin Lymphomas: Diffuse Large B-Cell and Follicular Subtypes

B-cell non-Hodgkin lymphomas (B-NHL), including diffuse large B-cell lymphoma (DLBCL) and follicular lymphoma (FL), have been leading indications for the development of multispecific antibodies. These malignancies frequently exhibit antigen heterogeneity and mechanisms of immune escape, contributing to relapse after conventional treatments. Multispecific platforms—including bispecific antibodies (BsAbs) and trispecific antibodies (TsAbs)—aim to address these limitations by redirecting immune effector cells and expanding antigenic coverage [57,58,59,60].

In DLBCL, BsAbs targeting CD20×CD3 have demonstrated robust clinical activity in relapsed/refractory (R/R) settings, including in patients previously treated with CAR-T therapies [57,58,59,60].

Glofitamab, utilizing a 2:1 CD20:CD3 configuration to enhance avidity, achieved an overall response rate (ORR) of 51.6% and complete response (CR) rate of 39.4% in a pivotal phase I/II study [61]. Similarly, mosunetuzumab demonstrated an ORR of 42% and CR rate of 23.9% in R/R DLBCL [62]. Epcoritamab, delivered subcutaneously, offers comparable efficacy and a favorable safety profile. In combination with lenalidomide, it achieved an overall response rate (ORR) of 75% and a complete response (CR) rate of 58% in patients with relapsed or refractory DLBCL, including those previously treated with CAR-T therapy [63].

In FL, BsAbs also offer a chemotherapy-free strategy for patients with limited options. In a pivotal phase II trial, mosunetuzumab achieved an ORR of 80.0% and a CR rate of 60.0% in patients with R/R disease after at least two prior therapies [64]. Epcoritamab, in combination with lenalidomide and rituximab (R2), has shown high response rates in early-phase trials and manageable toxicity [65]. These results have positioned BsAbs as viable alternatives to cytotoxic chemotherapy in indolent B-NHL.

To address clonal diversity and antigen loss in both DLBCL and FL, trispecific antibodies (TsAbs) are under active investigation. One such agent, PIT565 (CD19/CD3/CD2), demonstrated enhanced T-cell proliferation, cytokine production, and tumor lysis in preclinical models compared to bispecific antibodies, and showed sustained immune synapse formation and overcoming of T-cell exhaustion [66].

In the ongoing phase I dose-escalation trial (NCT05397496), PIT565 is being evaluated in patients with relapsed or refractory B-NHL and B-ALL after ≥2 prior lines of therapy, with dosing and safety profiles still under investigation. Additionally, a structurally optimized CD19/CD22/CD3 TsAb demonstrated 100% survival in murine xenograft models, superior to blinatumomab or combination bispecific antibodies, and effectively cleared leukemia in the presence of CD19/CD22 antigen heterogeneity, highlighting its ability to prevent immune escape [67,68].

In addition to antibody constructs, trispecific CAR-T cells, such as CAR20.19.22, are being explored to mitigate relapse through simultaneous targeting of multiple B-cell antigens. In a first-in-human phase I trial (NCT05418088) of patients with relapsed/refractory B-cell malignancies, complete response (CR) was achieved in 6 of 15 treated patients. Responses were observed in follicular lymphoma (FL), diffuse large B-cell lymphoma (DLBCL), mantle cell lymphoma (MCL), and B-ALL. Among nine patients with non-Hodgkin lymphoma (including three with Richter’s transformation), five achieved CR, with three remaining in remission beyond one year. This multi-antigen strategy aims to prevent immune escape due to single-antigen loss and is undergoing further investigation [69].

Altogether, BsAbs have already demonstrated meaningful efficacy across aggressive and indolent B-NHL subtypes, offering effective and accessible treatment options. TsAbs and trispecific CAR-T cell approaches represent the next frontier, with the potential to further deepen responses, delay relapse, and optimize immune targeting in biologically diverse lymphoma populations.

### 3.4. Multiple Myeloma (MM)

Multiple myeloma (MM) has become a central focus in the development of multispecific antibodies, particularly in the setting of triple-class refractory disease. BsAbs targeting B-cell maturation antigen (BCMA) have demonstrated transformative potential, offering off-the-shelf immune redirection for patients with few remaining options.

Among these, teclistamab, a BCMA × CD3 BsAb, has emerged as a frontrunner. In the pivotal MajesTEC-1 study, teclistamab achieved an overall response rate (ORR) of 63.0%, with a complete response (CR) or better in 39.4% of heavily pretreated patients [70]. Other BCMA-directed BsAbs, including elranatamab and linvoseltamab, have demonstrated comparable activity. In MagnetisMM-3, elranatamab monotherapy yielded an ORR of 61.0% and a CR rate of 31.7% [71]. In the LINKER-MM1 study, linvoseltamab achieved an ORR of 64% at the 200 mg dose, supporting its continued evaluation in phase III trials [72]. These data reinforce BCMA as a validated therapeutic target in relapsed/refractory MM. However, BCMA antigen loss and escape variants have led to the investigation of alternative targets.

One such target is GPRC5D, a receptor expressed on malignant plasma cells but not on normal B cells. Talquetamab, a GPRC5D × CD3 BsAb, has demonstrated high activity in patients refractory to prior anti-BCMA therapies. In the MonumenTAL-1 phase I/II trial, talquetamab achieved ORRs of 74.1% (400 µg/kg weekly) and 73.1% (800 µg/kg biweekly), with comparable efficacy observed across high-risk subgroups, including those with prior BCMA therapy [73]. Another target, FcRH5, has been leveraged in the development of cevostamab, which in an ongoing phase I study achieved an ORR of 54.5% at higher dose levels (132–198 mg), with observed durable responses in heavily pretreated MM patients [74]. These agents highlight the evolving role of BsAbs in expanding therapeutic coverage and overcoming resistance.

Building on these principles, trispecific antibodies (TsAbs) have been developed to further enhance immune engagement and durability of response. Several constructs incorporate both BCMA and GPRC5D, along with CD3, to broaden tumor recognition and prevent escape. For example, SIM0500, an IgG4-based TsAb targeting BCMA/GPRC5D/CD3, has demonstrated potent T-cell redirection and cytotoxicity against myeloma cells in preclinical models, including enhanced T-cell expansion, cytokine production, and lysis of tumor cells compared to bispecific formats. SIM0500 is currently undergoing evaluation in a first-in-human Phase I clinical trial in patients with relapsed/refractory multiple myeloma [75,76].

Co-stimulatory targeting represents another advancement in TsAb design. Constructs such as SAR442257, a CD38/CD3 × CD28 trispecific antibody, leverage CD28 signaling to promote robust T-cell activation and sustained anti-tumor responses. Preclinical studies demonstrated superior cytotoxicity compared to daratumumab and isatuximab, with SAR442257 reducing the viability of 10/10 tested multiple myeloma cell lines by 58–97% within 24 h, and significantly enhancing T-cell–mediated killing in autologous co-cultures from relapsed/refractory MM (RRMM) patients. The antibody was designed using a Cross-Over Dual Variable (CODV) IgG4 format with Fc mutations to avoid cytokine release. Importantly, SAR442257 is currently under clinical evaluation in a phase I trial (NCT04401020) for RRMM and non-Hodgkin lymphoma [77].

Similarly, dual-target TsAbs engaging CD3/CD28 and a tumor-associated antigen (TAA) are being developed to amplify immune responses in MM by co-engaging T-cell activation and tumor specificity in a single construct [78].

**Figure 2:**
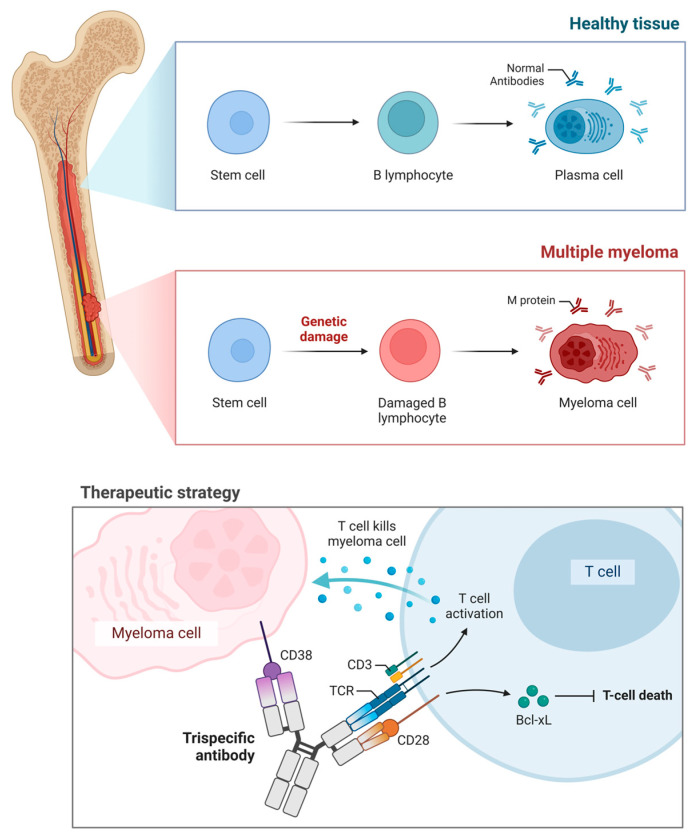
Immunotherapy in multiple myeloma—demonstrating trispecific engagement pathways.

Altogether, BsAbs have already begun reshaping the treatment algorithm in relapsed/refractory MM by delivering deep responses across antigen targets. TsAbs offer a logical and mechanistically potent extension of this platform—capable of improving response depth, overcoming resistance via dual antigen engagement, and potentiating T-cell activity through co-stimulation. As clinical trials mature, these agents may establish a new paradigm for immune-based therapy in multiple myeloma (Figure 2).

## 4. Immune-Related Toxicities of Bispecific and Trispecific Antibodies

Multispecific antibodies—including bispecific (BsAbs) and trispecific antibodies (TsAbs)—have significantly advanced treatment in hematologic malignancies by harnessing immune effector cells for targeted cytotoxicity. However, their potent immunostimulatory activity also gives rise to unique and sometimes severe toxicities. These adverse events primarily stem from T-cell activation and cytokine release, with trispecific constructs potentially amplifying these effects through co-stimulatory signaling or dual-antigen targeting. While most toxicities are manageable with early recognition and appropriate supportive care, their occurrence necessitates vigilant monitoring in clinical practice. The following sections summarize key toxicities associated with BsAbs and TsAbs, including cytokine release syndrome (CRS), neurotoxicity, cytopenias, infections, and on-target off-tumor effects, along with current management strategies

### 4.1. Cytokine Release Syndrome (CRS)

Cytokine release syndrome (CRS) is the most frequently reported immune-related adverse event associated with BsAbs. It arises from robust immune activation—particularly T-cell engagement—and the subsequent release of proinflammatory cytokines, including interleukin-6 (IL-6), IL-2, and tumor necrosis factor-alpha (TNF-α). Clinically, CRS typically manifests within hours to days following treatment initiation, presenting with fever, hypotension, hypoxia, tachycardia, and, in severe cases, multiorgan dysfunction [79,80,81,82].

The incidence of CRS is construct- and platform-dependent. In the MajesTEC-1 trial of teclistamab, CRS occurred in 72.1% of patients, although most events were low grade (≥Grade 3: 0.6%) [83]. Glofitamab similarly demonstrated a CRS rate of 50.3%, with ≥Grade 3 events in 3.5% [84]. Subcutaneous BsAb formulations, such as epcoritamab, have been associated with lower peak cytokine levels and may reduce CRS severity compared to intravenous delivery [85]. As TsAbs incorporate additional activating domains (e.g., CD28), they may carry a higher theoretical risk of CRS, although clinical data are still emerging [86].

Management of CRS is guided by the ASTCT consensus grading criteria and involves a stepwise approach. Mild cases are treated with supportive care, including antipyretics, intravenous fluids, and supplemental oxygen. Tocilizumab, an IL-6 receptor antagonist, remains the first-line treatment for moderate to severe CRS and typically results in rapid symptom improvement [87]. Corticosteroids may be used in steroid-refractory cases or when neurologic symptoms co-occur. Additional mitigation strategies include step-up dosing, premedication with corticosteroids or antihistamines, and, for investigational constructs, incorporation of cytokine attenuation designs such as IL-6R-targeted elements (e.g., TriTECM platforms) [88,89].

As clinical experience with TsAbs expands, further refinements in construct design and risk-adapted management are anticipated to reduce CRS incidence while preserving therapeutic efficacy (Table 1).

### 4.2. Immune Effector Cell-Associated Neurotoxicity Syndrome (ICANS)

Immune effector cell-associated neurotoxicity syndrome (ICANS) is a recognized complication of T cell-engaging immunotherapies, including both bispecific (BsAbs) and trispecific antibodies (TsAbs). ICANS is believed to result from cytokine-mediated blood–brain barrier disruption and endothelial activation. Symptoms typically include confusion, aphasia, tremors, and, in severe cases, seizures or cerebral edema [90,91].

The incidence of ICANS varies across constructs and settings. In the MajesTEC-1 trial, teclistamab was associated with ICANS in 3.0% of patients, with ≥Grade 3 events in 0.6% [92]. However, a recent multi-institutional real-world study reported a higher ICANS incidence of 11%, including Grade ≥3 events in 4.6% of patients [93]. Glofitamab trials have reported ICANS in 5.3% of patients [94]. Among BsAbs, the highest rates have been observed with blinatumomab, where ICANS occurred in 9–13% of patients, including ≥Grade 3 events in up to 10% [95,96]. Talquetamab, a GPRC5D × CD3 bispecific antibody, also showed ICANS in 10.7% to 11.0% of patients in clinical trials, with most events being low grade and often occurring in the context of concurrent cytokine release syndrome (CRS) [97].

For trispecific constructs, comprehensive ICANS data remain limited, but the inclusion of co-stimulatory elements or multiple antigen targets may amplify the neurotoxicity risk. Ongoing research is focused on characterizing the neurologic safety profile of emerging trispecific formats [98].

Management of ICANS follows the ASTCT consensus guidelines. Mild cases are typically monitored without intervention, while moderate to severe events are managed with corticosteroids. Tocilizumab is not effective for isolated ICANS but may be used when CRS co-occurs. Prophylactic anticonvulsants (e.g., levetiracetam) are often employed in high-risk patients [99].

Future mitigation strategies may include step-up dosing, route modifications, and molecular designs that modulate cytokine release (e.g., TriTECM constructs incorporating IL-6R targeting) [100]. As experience with multispecific antibodies grows, tailored prevention and management approaches will be essential to balance efficacy and safety.

### 4.3. Hematologic Toxicities

Hematologic toxicities—including neutropenia, anemia, and thrombocytopenia—are common adverse effects associated with both bispecific (BsAbs) and trispecific antibodies (TsAbs). These toxicities primarily result from on-target, off-tumor effects on normal hematopoietic cells, particularly when the target antigen (e.g., CD19, BCMA, CD123) is also expressed on B cells, plasma cells, or progenitor populations [101].

In the MajesTEC-1 study of teclistamab, a BCMA × CD3 bispecific antibody, Grade ≥3 neutropenia was reported in 51.1% of patients, anemia in 37.8%, and thrombocytopenia in 21.5% [102]. These cytopenias are particularly problematic in heavily pretreated patients, where they contribute to increased risks of infection, bleeding, and treatment delays. Real-world data corroborate these findings, indicating that hematologic toxicities remain a significant concern in clinical practice [103].

Trispecific antibodies targeting shared antigens such as BCMA or CD123, or co-targeting multiple domains, are currently under early clinical investigation. Comprehensive clinical data specifically evaluating hematologic toxicities of trispecific antibodies in hematologic malignancies remain limited. Early clinical experience with trispecific antibodies in multiple myeloma, such as ISB 2001, has so far shown manageable safety signals, although detailed incidence rates and long-term hematologic toxicity profiles are still emerging [104,105,106,107].

While current management strategies for hematologic toxicities are predominantly extrapolated from clinical experience with bispecific antibodies, ongoing and future clinical trials will be essential to establish evidence-based approaches specifically optimized for trispecific antibody platforms. As trispecific therapies are introduced into earlier lines of therapy and into less heavily pretreated patient cohorts, the proactive identification and mitigation of hematologic toxicities will become increasingly critical to preserving patient quality of life, minimizing treatment interruptions, and ensuring therapeutic continuity.

### 4.4. Infections

Infections represent a common and potentially serious complication associated with multispecific antibody therapies, particularly BsAbs and emerging TsAbs that target B-lineage or plasma cell antigens. The infection risk is multifactorial, arising from a combination of immune effector cell engagement, B-cell and plasma cell depletion, therapy-induced hypogammaglobulinemia, and cytopenias, particularly neutropenia [108,109]. Together, these immunologic disruptions contribute to an increased susceptibility to bacterial, viral, fungal, and opportunistic infections.

In the MajesTEC-1 trial, teclistamab—a BCMA × CD3 bispecific antibody—was associated with infections in 76.4% of treated patients, including Grade ≥3 infections in 44.8% [102,110]. Similarly, clinical trials evaluating other bispecific constructs such as glofitamab and mosunetuzumab reported serious infections in approximately 15–20% of patients [111,112]. These findings underscore the significant infectious risks that accompany multispecific antibody-mediated immune modulation, largely attributed to sustained B-cell depletion and resulting hypogammaglobulinemia.

Experience with trispecific antibodies in hematologic malignancies remains limited, and robust clinical data specifically characterizing infection risk are still emerging. Early-phase trials, including the evaluation of MBS314—a GPRC5D × BCMA × CD3 trispecific antibody—have reported hematologic toxicities such as Grade 3 lymphopenia and leukopenia [113], both recognized as important predisposing factors for infectious complications. Although no severe infections were documented within this preliminary cohort, these observations highlight the necessity for vigilant infection surveillance in patients receiving trispecific therapies.

Hypogammaglobulinemia, a key driver of infection susceptibility, has been well documented across bispecific antibody (BsAb) platforms, particularly in constructs targeting BCMA and CD19. Although direct clinical data are currently lacking for TsAbs, similar theoretical risks exist, given their shared targeting of plasma cell antigens such as BCMA and GPRC5D [114,115].

Management of infection risk in patients treated with multispecific antibodies requires a proactive and individualized approach. Baseline assessment of immunoglobulin levels is recommended prior to therapy initiation, and intravenous immunoglobulin (IVIG) replacement should be considered for patients with symptomatic hypogammaglobulinemia. Prophylactic antimicrobial strategies—including antibacterial, antiviral, and antifungal agents—should be tailored according to patient-specific risk factors, severity of cytopenias, and history of prior infections. Immunization against encapsulated bacteria (e.g., *Streptococcus pneumoniae*) and seasonal respiratory viruses (e.g., influenza, SARS-CoV-2) should be administered before treatment initiation whenever feasible. Furthermore, close monitoring for early signs of infection, coupled with prompt initiation of empiric antimicrobial therapy at symptom onset, is critical to minimizing infectious morbidity and mortality [116,117].

As the clinical development of trispecific antibodies progresses, prospective studies will be essential to comprehensively define infection risks across different constructs, particularly in hematologic malignancies.

## 5. Resistance Mechanisms and Therapeutic Strategies in T-Cell Redirecting Bispecific and Trispecific Antibody Therapy

The clinical success of T-cell redirecting BsAbs and TsAbs in hematologic malignancies, particularly in relapsed or refractory multiple myeloma, has been tempered by the development of both primary and acquired resistance. Resistance mechanisms are diverse and involve tumor-intrinsic alterations, dysfunction of immune effector cells, and immunosuppressive changes within the tumor microenvironment. A detailed understanding of these biological processes is critical for the optimization of therapeutic efficacy, the development of predictive biomarkers, and the design of rational strategies to prevent or overcome treatment failure.

###  5.1. Primary and Secondary Resistance

Primary resistance, defined as the failure to achieve an initial clinical response, has been associated with several factors, including high tumor burden, impaired baseline T-cell function, and the presence of an immunosuppressive bone marrow environment [118]. Patients with high levels of regulatory T cells, exhausted T-cell phenotypes, and elevated soluble antigen levels at baseline are more likely to experience primary resistance [118].

In contrast, secondary resistance, defined as relapse after an initial response, commonly arises through mechanisms such as antigen escape, progressive T-cell dysfunction, and remodeling of the tumor microenvironment [118,119]. Tumor-intrinsic mechanisms contributing to secondary resistance include biallelic deletions or mutations affecting key target antigens, such as TNFRSF17 (BCMA) and GPRC5D [119]. Loss of surface antigen expression through genetic or epigenetic mechanisms has been documented in patients relapsing after BsAb or potentially in TsAb therapy [119].

In addition, chronic T-cell stimulation induced by prolonged exposure to T-cell–redirecting therapies promotes functional T-cell exhaustion, characterized by upregulation of inhibitory receptors such as PD-1, LAG-3, and TIM-3, ultimately impairing cytotoxic responses [120]. These mechanisms often act in concert with microenvironmental factors, including expansion of immunosuppressive myeloid-derived suppressor cells and regulatory T cells, further diminishing T-cell efficacy [120].

###  5.2. Biomarkers: Pretreatment and On-Treatment

Identifying predictive biomarkers of response or resistance is critical to guide patient selection and therapeutic sequencing. Pretreatment factors associated with favorable response include a high frequency of circulating naïve and central memory CD8+ T cells, low baseline levels of soluble target antigens such as soluble BCMA (sBCMA) [121], and the absence of genomic abnormalities involving key target genes [122]. Conversely, high tumor burden, elevated T-cell exhaustion markers, and genomic alterations such as heterozygous deletions of TNFRSF17 or GPRC5D at baseline have been associated with inferior outcomes [122,123,124].

During therapy, the emergence of antigen loss, either through genomic alterations or epigenetic silencing, can be detected by flow cytometry or molecular profiling and correlates with clinical relapse [122]. Longitudinal immune monitoring has revealed that progressive upregulation of exhaustion markers on T cells during BsAb treatment precedes clinical disease progression [125,126]. These findings highlight the importance of dynamic biomarker assessment, both before and during therapy, to anticipate and address evolving resistance mechanisms.

### 5.3. Minimal Residual Disease (MRD)

Minimal residual disease (MRD) has emerged as a pivotal prognostic marker in multiple myeloma, offering insights into treatment efficacy and long-term outcomes. Achieving MRD negativity is associated with significantly improved progression-free survival and overall survival, surpassing traditional response criteria such as complete response [127].

In the context of bispecific antibody therapies, MRD assessment has demonstrated notable prognostic value. In the MajesTEC-1 trial evaluating teclistamab, MRD negativity at a sensitivity threshold of 10^−5^ was achieved in a substantial proportion of patients, and sustained MRD negativity correlated with prolonged remissions [128]. Similarly, in early-phase studies involving elranatamab, MRD negativity has been consistently associated with deeper and more durable responses [129].

The prognostic significance of MRD extends beyond the bispecific antibody setting. Large prospective trials, including those from the GEM/PETHEMA and IFM groups, have shown that MRD-negative status predicts superior outcomes, independent of the therapeutic modality used. MRD negativity has been validated as a surrogate endpoint for progression-free survival and is increasingly incorporated into clinical trial designs evaluating novel multispecific antibody therapies [130].

Moreover, MRD assessment is informing evolving strategies regarding treatment duration and intensity. Fixed-duration treatment approaches guided by MRD status are under investigation, with the goal of minimizing long-term toxicity while preserving efficacy. Early discontinuation of therapy in patients achieving sustained MRD negativity, as well as preemptive interventions upon MRD resurgence, represent important future directions in the field [131].

Overall, MRD negativity serves not only as a critical measure of treatment depth but also as an emerging tool to personalize therapy and optimize long-term outcomes in patients receiving BsAb and TsAb therapies.

### 5.4. Retreatment and Approaches to Overcome Resistance

Efforts to overcome resistance after failure of bispecific or trispecific antibody therapy have focused on multiple complementary strategies. Switching to alternative antigen targets, such as transitioning from BCMA-directed therapies to GPRC5D- or FcRH5-directed constructs, has demonstrated clinical efficacy in the setting of antigen loss in multiple myeloma and is similarly being explored with CD19- and CD20-directed therapies in B-cell malignancies [132,133]. Combination approaches, including the concurrent use of two bispecific antibodies targeting distinct tumor-associated antigens or the administration of trispecific constructs engaging CD3 and two tumor antigens, have shown promise in mitigating antigen escape and broadening immune engagement [132,134].

Augmenting T-cell fitness through combination therapies is an additional strategy under active investigation. The addition of immune checkpoint inhibitors, particularly PD-1 or PD-L1 blockade, has demonstrated the ability to restore T-cell functionality and enhance bispecific antibody efficacy in preclinical and early clinical studies across lymphoid malignancies [135]. Similarly, incorporation of cereblon E3 ligase modulators (CELMoDs) such as iberdomide and mezigdomide has been shown to potentiate T-cell activation and immune synapse formation, synergizing with T-cell-engaging bispecifics in both multiple myeloma and lymphoma settings [136].

Construct optimization, including the development of bispecific antibodies with attenuated CD3-binding affinity, aims to reduce cytokine release, limit T-cell exhaustion, and improve pharmacokinetic properties without compromising antitumor activity [137]. Additionally, fixed-duration treatment strategies, exemplified by cevostamab trials in multiple myeloma and by limited duration bispecific antibody regimens in diffuse large B-cell lymphoma, have demonstrated that durable remissions are achievable without continuous exposure, potentially allowing immune recovery and reducing cumulative toxicity [133,138].

As the understanding of resistance biology deepens, rational therapeutic strategies incorporating antigen switching, immune modulation, and optimized construct design will be essential to sustain durable responses in patients treated with bispecific and trispecific antibody therapies.

## 6. Future Directions and Strategic Positioning of Bispecific and Trispecific Antibodies in Hematologic Malignancies

The landscape of bispecific (BsAbs) and trispecific antibodies (TsAbs) in hematologic malignancies is evolving rapidly, transitioning beyond proof-of-concept into disease-specific integration, earlier lines of therapy, and combination regimens. As clinical experience matures, novel molecular designs, expansion into solid tumors, and incorporation of artificial intelligence (AI)–based strategies are poised to further broaden the impact of multispecific immunotherapies.

### 6.1. Disease-Specific Integration and Sequencing

In DLBCL, BsAbs such as glofitamab, mosunetuzumab, and epcoritamab have been incorporated into the relapsed/refractory setting, offering chemotherapy-free, off-the-shelf options [139]. Early studies support their use in fixed-duration regimens, and ongoing trials are investigating combination approaches to enhance depth and durability of response. Trispecific constructs, such as CD19/CD22/CD3 formats [140] and trispecific CAR-T cell approaches [141], are under evaluation to address clonal heterogeneity and antigen escape, particularly in high-risk patients and those relapsing after CAR-T cell therapy.

In FL, BsAbs have demonstrated robust efficacy in heavily pretreated patients. Mosunetuzumab and epcoritamab have achieved high response rates, offering a potential paradigm shift toward immune-based therapies without chemotherapy. Although trispecific constructs are not yet in widespread clinical use for FL, they are being developed to preempt clonal evolution and to overcome genetic diversity, particularly in transformation-prone disease [139,140].

In MM, BsAbs targeting BCMA, such as teclistamab, and GPRC5D, such as talquetamab, have achieved deep and durable responses in triple-class refractory settings [142,143]. TsAbs incorporating BCMA/GPRC5D/CD3 or co-stimulatory domains such as CD28 or 4-1BB are emerging to enhance T-cell persistence, mitigate antigen escape, and improve durability [144]. Their potential application in early relapse or first salvage settings, particularly among patients with impaired T-cell fitness, represents a major future direction.

### 6.2. Beyond Trispecifics: New Multispecific Designs

Advancements beyond traditional trispecific formats are actively being pursued to enhance the efficacy and safety of multispecific antibodies. One such innovation is the development of tetrafunctional constructs, exemplified by the TriTECM platform. These constructs integrate T-cell engagement with cytokine attenuation mechanisms, aiming to reduce CRS while preserving anti-tumor efficacy. Specifically, TriTECM molecules combine T-cell redirection with interleukin-6 receptor (IL-6R) blockade, demonstrating attenuated T-cell activation and reduced interferon-gamma production, which suggests potential for mitigating CRS in clinical settings [145].

Another emerging strategy involves checkpoint-integrated multispecific antibodies that simultaneously target tumor antigens and inhibitory pathways such as PD-1 or TIGIT. These constructs are designed to enhance T-cell activation within the tumor microenvironment by concurrently blocking multiple immune checkpoints, thereby overcoming resistance mechanisms associated with monotherapies [146].

Additionally, protease-cleavable masking constructs have been engineered to restrict antibody activation specifically to the tumor microenvironment. By incorporating tumor-specific protease-sensitive linkers, these antibodies remain inactive in systemic circulation and are activated upon encountering the protease-rich tumor milieu. This approach aims to minimize off-target effects and systemic toxicity, enhancing the therapeutic index of multispecific antibodies [147].

Collectively, these engineering efforts focus on achieving more selective tumor targeting, improved pharmacokinetics, and superior immune engagement while minimizing off-target effects. These innovations represent a critical next phase in the evolution of multispecific therapies, potentially addressing limitations observed with current bispecific and trispecific antibodies.

### 6.3. Expanding into Solid Tumors

BsAbs and TsAbs, initially validated in hematologic malignancies, are now demonstrating early clinical efficacy in solid tumors. Constructs targeting well-defined tumor-associated antigens such as HER2, DLL3, PSMA, CEA, and EGFRvIII have entered advanced clinical trials across a variety of solid tumor types, including breast cancer, small cell lung cancer (SCLC), prostate cancer, and glioblastoma [148,149,150,151].

Several BsAbs have already reached human testing. Catumaxomab, targeting EpCAM and CD3, was the first bispecific antibody approved for malignant ascites, although later withdrawn for commercial reasons [148]. Modern BiTEs, such as tarlatamab (DLL3 × CD3) in SCLC [149], pasotuxizumab (PSMA × CD3) in prostate cancer [150], and etevritamab (EGFRvIII × CD3) in glioblastoma [151], have demonstrated antitumor activity and manageable toxicity profiles in early-phase trials. Tarlatamab, notably, has shown partial responses and stable disease rates exceeding 40% in SCLC patients, marking a significant advance [149].

However, significant challenges remain. Solid tumors pose barriers such as limited T-cell infiltration, immunosuppressive tumor microenvironments, and higher risk of on-target, off-tumor toxicity [152]. Strategies to address these challenges include tumor-selective activation through protease-cleavable masking domains [152], development of half-life-extended (HLE) BiTEs to optimize pharmacokinetics [153], and incorporation of local activation features that restrict T-cell engagement to the tumor microenvironment [152].

Thus, while multispecific antibody therapies for solid tumors are still maturing, early results are encouraging. The authors believe that incorporating tumor-penetrating strategies into bispecific antibody (BsAb) platforms represents a critical advancement toward expanding their efficacy beyond hematologic malignancies. While BsAbs have demonstrated impressive activity in bone marrow-localized disease, their therapeutic potential in solid tumors—and in mass-forming plasma cell neoplasms such as plasmacytomas—has been limited in part by poor tissue penetration and immune exclusion.

Recent engineering approaches, including the development of smaller BsAb formats (e.g., DARTs, nanobody-based constructs), fusion to extracellular matrix-degrading enzymes, and chemokine-modulated T-cell recruitment, offer a rational path to overcoming these barriers. We believe that such innovations are particularly relevant for high-risk myeloma subtypes with extramedullary or plasmacytoma-predominant disease, where standard BsAbs may have reduced efficacy due to physical and microenvironmental constraints. These approaches warrant further clinical evaluation as a strategy to broaden BsAb applicability in both solid tumors and solid-predominant hematologic malignancies.

### 6.4. Artificial Intelligence in Multispecific Antibody Development

Artificial intelligence (AI) and machine learning (ML) are increasingly integral to the design, development, and deployment of multispecific antibodies. AI algorithms are being utilized to identify optimal antigen pairs or triplets based on multi-omic datasets, including single-cell transcriptomic and proteomic profiles [154].

Furthermore, explainable AI (XAI) frameworks, particularly those leveraging SHapley Additive exPlanations (SHAP), are providing actionable insights into predicting patient response, toxicity risks, and optimal combination strategies. SHAP-based models have been used to interpret immunotherapy outcomes by identifying individual patient features that drive response or resistance, including baseline T-cell profiles, tumor burden, and cytokine dynamics. Specifically, for BsAb therapies, SHAP modeling has been applied to predict the likelihood and severity of CRS and ICANS based on early cytokine measurements and patient-specific immunologic signatures [155,156]. These applications allow dynamic risk stratification and may guide early intervention strategies, such as step-up dosing or prophylactic immunosuppression, thereby improving the therapeutic index of BsAbs and emerging multispecific constructs.

AI-guided models are also being developed to dynamically adjust dosing and therapeutic sequencing in real time based on evolving biomarkers. Advances in AI-driven antibody engineering platforms are enabling the large-scale analysis of antigen–antibody interactions, optimization of multispecific constructs, and prediction of pharmacologic behavior across different tumor microenvironments [157].

The integration of AI into multispecific antibody development and clinical deployment holds the promise of greater precision, personalization, and efficiency in future immunotherapy paradigms, bridging the gap between complex molecular constructs and individualized patient care.

## 7. Conclusions

BsAbs and TsAbs have fundamentally reshaped the immunotherapeutic landscape in hematologic malignancies, offering transformative advances in disease control across multiple settings. By leveraging dual or triple antigen targeting, co-stimulatory integration, and molecular engineering innovations, these constructs have overcome significant barriers such as antigen escape, immune exhaustion, and treatment resistance. The clinical success of BsAbs in diseases such as acute lymphoblastic leukemia, diffuse large B-cell lymphoma, follicular lymphoma, and multiple myeloma has validated T-cell redirection as a potent therapeutic strategy, while early-phase data on TsAbs and emerging multispecific formats indicate even greater potential for immune engagement and durability of response.

Nevertheless, challenges remain. Resistance mechanisms, immune-related toxicities such as CRS and ICANS, and infection risks necessitate ongoing innovation in construct design, patient selection, and supportive care strategies. Advances such as tetrafunctional constructs, tumor microenvironment-restricted activation, and checkpoint-integrated multispecific antibodies aim to enhance the safety and efficacy of these agents.

Importantly, the field is poised for broader expansion beyond hematology into solid tumors, driven by promising early results targeting antigens such as HER2, DLL3, PSMA, and EGFRvIII. Furthermore, the integration of artificial intelligence (AI) and machine learning (ML) into multispecific antibody development is opening new frontiers in target discovery, biomarker-driven personalization, toxicity prediction, and real-time therapeutic adaptation.

Together, these advances herald a new era in immune-based therapy. BsAbs and TsAbs are no longer experimental concepts but integral components of precision oncology, with the potential to redefine treatment paradigms across both hematologic and solid tumors. Continued clinical translation, interdisciplinary innovation, and strategic integration into multimodal treatment frameworks will be critical to fully realize their transformative potential.

## Figures and Tables

**Table 1:** Summary of bispecific and trispecific antibodies in hematologic malignancies.

Disease	Agent	Target(s)	Type	Phase/Approval	ORR/CR (%)	Notable Comments
ALL	Blinatumomab	CD19 × CD3	BsAb (BiTE)	FDA/EMA Approved (R/R, MRD)	ORR: ~44%; CR: ~19%	Approved for Ph-negative ALL; MRD clearance indication.
ALL	CD19/CD22/CD3	CD19, CD22, CD3	TsAb	Preclinical	N/A	Dual-antigen targeting to overcome CD19 escape.
AML	Flotetuzumab	CD123 × CD3	BsAb (DART)	Phase I/II	ORR: 24%	Investigated in R/R AML; TP53-mutated cohort notable.
AML	CLEC12A/CD16/IL-15 (TriKE)	CLEC12A, CD16, IL-15	TriKE	Preclinical	N/A	NK-cell engagement plus cytokine-driven proliferation.
DLBCL	Glofitamab	CD20 × CD3	BsAb	EMA Approved (Post-CAR-T R/R LBCL)	ORR: 51.6%; CR: 39.4%	Step-up dosing improves safety and durability.
DLBCL	Mosunetuzumab	CD20 × CD3	BsAb	EMA Approved (FL); Phase II (DLBCL)	ORR: 43.2%; CR: 24.8%	Approved for FL; active investigation for DLBCL.
DLBCL	CAR20.19.22	CD20, CD19, CD22	Tri-CAR	Phase I ongoing	ORR: 75%; CR: 42%	Multitarget CAR-T; antigen escape prevention.
FL	Mosunetuzumab	CD20 × CD3	BsAb	EMA Approved (FL)	ORR: 80%; CR: 60%	First bispecific approved for FL (chemo-free option).
MM	Teclistamab	BCMA × CD3	BsAb	FDA/EMA Approved	ORR: 63%; CR: ≥39.4%	First BsAb for RRMM; subcutaneous administration.
MM	Talquetamab	GPRC5D × CD3	BsAb	FDA Approved (post–BCMA)	ORR: ~73%	Targets GPRC5D; dermatologic toxicity common.
MM	SIM0500	BCMA/GPRC5D/CD3	TsAb	Phase I ongoing	N/A	Dual-antigen engagement to prevent BCMA escape.
MM	SAR442257	CD38/CD28 × CD3	TsAb	Phase I ongoing	N/A	CD28-mediated T-cell co-stimulation; early-phase data.

## References

[1] Moreau P., Touzeau C. (2022). T-cell–redirecting bispecific antibodies in multiple myeloma: A revolution?. Blood J. Am. Soc. Hematol..

[2] Kordic A., Phillips T.J., Weiss J. (2025). The Current State of Bispecific Antibodies and T-Cell Directed Therapy in NHL. Cancers.

[3] Castaneda-Puglianini O., Chavez J.C. (2021). Bispecific antibodies for non-Hodgkin’s lymphomas and multiple myeloma. Drugs Context.

[4] Lantz J., Pham N., Jones C., Reed D., El Chaer F., Keng M. (2024). Blinatumomab in Practice. Curr. Hematol. Malig. Rep..

[5] Chakraborty R., Cheruvalath H., Patwari A., Szabo A., Schinke C., Dhakal B., Lentzsch S., D’Souza A., Mohyuddin G.R., Julian K. (2024). Sustained remission following finite duration bispecific antibody therapy in patients with relapsed/refractory myeloma. Blood Cancer J..

[6] Li J., Slaga D., Johnston J., Junttila T.T. (2023). IMiDs Augment CD3-Bispecific Antibody–Induced CD8+ T-Cell Cytotoxicity and Expansion by Enhancing IL2 Production. Mol. Cancer Ther..

[7] Baines A.C., Kanapuru B., Zhao J., Price L.S., Zheng N., Konicki R., Manning M.L., Gehrke B.J., Theoret M.R., Gormley N.J. (2024). FDA Approval Summary: Teclistamab–A Bispecific CD3 T-Cell Engager for Patients with Relapsed or Refractory Multiple Myeloma. Clin. Cancer Res..

[8] Lancman G., Richter J., Chari A. (2020). Bispecifics, trispecifics, and other novel immune treatments in myeloma. Hematol. Am. Soc. Hematol. Educ. Program.

[9] Qi X., Chen G., Cao Y.J. (2025). Optimal Structural Designs of Trispecific Antibodies to Enhance Therapeutic Efficacy in Solid Tumors and Hematological Malignancies. Cancer Immunosurveill. Methods Protoc..

[10] Schoenfeld K., Harwardt J., Kolmar H. (2024). Better safe than sorry: Dual targeting antibodies for cancer immunotherapy. Biol. Chem..

[11] Velasquez M.P., Bonifant C.L., Gottschalk S. (2018). Redirecting T cells to hematological malignancies with bispecific antibodies. Blood J. Am. Soc. Hematol..

[12] Restelli C., Ruella M., Paruzzo L., Tarella C., Pelicci P.G., Colombo E. (2024). Recent advances in immune-based therapies for acute myeloid leukemia. Blood Cancer Discov..

[13] van de Donk N.W., O’Neill C., de Ruijter M.E., Verkleij C.P., Zweegman S. (2023). T-cell redirecting bispecific and trispecific antibodies in multiple myeloma beyond BCMA. Curr. Opin. Oncol..

[14] Godfrey J.K., Gao L., Shouse G., Song J.Y., Pak S., Lee B., Chen B.T., Kallam A., Baird J.H., Marcucci G. (2024). Glofitamab stimulates immune cell infiltration of CNS tumors and induces clinical responses in secondary CNS lymphoma. Blood.

[15] Sehn L.H., Bartlett N.L., Matasar M.J., Schuster S.J., Assouline S.E., Giri P., Kuruvilla J., Shadman M., Cheah C.Y., Dietrich S. (2025). Long-term 3-year follow-up of mosunetuzumab in relapsed or refractory follicular lymphoma after≥ 2 prior therapies. Blood.

[16] Thieblemont C., Karimi Y.H., Ghesquieres H., Cheah C.Y., Clausen M.R., Cunningham D., Jurczak W., Do Y.R., Gasiorowski R., Lewis D.J. (2024). Epcoritamab in relapsed/refractory large B-cell lymphoma: 2-year follow-up from the pivotal EPCORE NHL-1 trial. Leukemia.

[17] Qin X., Ning W., Liu H., Liu X., Luo W., Xia N. (2024). Stepping forward: T-cell redirecting bispecific antibodies in cancer therapy. Acta Pharm. Sin. B.

[18] Fleury I., MacDonald D., Shafey M., Christofides A., Sehn L.H. (2025). Optimal Use of Bispecific Antibodies for the Treatment of Diffuse Large B-Cell Lymphoma in Canada. Curr. Oncol..

[19] Jain T., Litzow M.R. (2020). Management of toxicities associated with novel immunotherapy agents in acute lymphoblastic leukemia. Ther. Adv. Hematol..

[20] Cho S.F., Lin L., Xing L., Wen K., Yu T., Wahl J., Matthes K., Munshi N., Anderson K.C., Arvedson T. (2019). AMG 701, a half-life extended anti-BCMA BiTE^®^, potently induces T cell-redirected lysis of human multiple myeloma cells and can be combined with IMiDs to overcome the immunosuppressive bone marrow microenvironment. Clin. Lymphoma Myeloma Leuk..

[21] Harrison S.J., Minnema M.C., Lee H.C., Spencer A., Kapoor P., Madduri D., Larsen J., Ailawadhi S., Kaufman J.L., Raab M.S. (2020). A phase 1 first in human (FIH) study of AMG 701, an anti-B-cell maturation antigen (BCMA) half-life extended (HLE) BiTE®(bispecific T-cell engager) molecule, in relapsed/refractory (RR) multiple myeloma (MM). Blood.

[22] Shirley M. (2023). Glofitamab: First approval. Drugs.

[23] Minson A., Dickinson M. (2021). Glofitamab CD20-TCB bispecific antibody. Leuk. Lymphoma.

[24] Nooka A.K., Rodriguez C., Mateos M.V., Manier S., Chastain K., Banerjee A., Kobos R., Qi K., Verona R., Doyle M. (2024). Incidence, timing, and management of infections in patients receiving teclistamab for the treatment of relapsed/refractory multiple myeloma in the MajesTEC-1 study. Cancer.

[25] Moreau P., van de Donk N.W., Delforge M., Einsele H., De Stefano V., Perrot A., Besemer B., Pawlyn C., Karlin L., Manier S. (2023). Comparative efficacy of teclistamab versus current treatments in real-world clinical practice in the prospective LocoMMotion study in patients with triple-class-exposed relapsed and/or refractory multiple myeloma. Adv. Ther..

[26] Dunai C., Ames E., Ochoa M.C., Fernandez-Sendin M., Melero I., Simonetta F., Baker J., Alvarez M. (2022). Killers on the loose: Immunotherapeutic strategies to improve NK cell-based therapy for cancer treatment. Int. Rev. Cell Mol. Biol..

[27] Page A., Chuvin N., Valladeau-Guilemond J., Depil S. (2024). Development of NK cell-based cancer immunotherapies through receptor engineering. Cell. Mol. Immunol..

[28] Sun Y., Zhou L., Gu X., Zhao J., Bi J., Pan L. (2025). Leveraging T cell co-stimulation for enhanced therapeutic efficacy of trispecific antibodies targeting prostate cancer. J. Immunother. Cancer.

[29] Sandeep Shinde S.H., Ahmed S., Sharma S.S., Pande A.H. (2024). Engineered polyspecific antibodies: A new frontier in the field of immunotherapeutics. Immunology.

[30] Thisted T., Smith F.D., Jiang Z.G., Onumajuru A., Biesova Z., Kleschenko Y., Malhotra K., Saxena V., Mukherjee A., van der Horst E.H. (2025). Dual Targeting for Enhanced Tumor Immunity: Conditionally Active CD28xVISTA Bispecific Antibodies Promote Myeloid-Driven T-Cell Activation. bioRxiv.

[31] Lotze M.T., Olejniczak S.H., Skokos D. (2024). CD28 co-stimulation: Novel insights and applications in cancer immunotherapy. Nat. Rev. Immunol..

[32] In H., Park M., Lee H., Han K.H. (2025). Immune Cell Engagers: Advancing Precision Immunotherapy for Cancer Treatment. Antibodies.

[33] Klingemann H. (2023). The NK-92 cell line—30 years later: Its impact on natural killer cell research and treatment of cancer. Cytotherapy.

[34] Maskalenko N.A., Zahroun S., Tsygankova O., Anikeeva N., Sykulev Y., Campbell K.S. (2025). The FcγRIIIA (CD16) L48-H/R Polymorphism Enhances NK Cell–Mediated Antibody-Dependent Cellular Cytotoxicity by Promoting Serial Killing. Cancer Immunol. Res..

[35] Tapia-Galisteo A., Compte M., Álvarez-Vallina L., Sanz L. (2023). When three is not a crowd: Trispecific antibodies for enhanced cancer immunotherapy. Theranostics.

[36] Zhao L., Li S., Wei X., Qi X., Liu D., Liu L., Wen F., Zhang J.S., Wang F., Liu Z.L. (2022). A novel CD19/CD22/CD3 trispecific antibody enhances therapeutic efficacy and overcomes immune escape against B-ALL. Blood J. Am. Soc. Hematol..

[37] Kuchnio A., Yang D., Vloemans N., Lowenstein C., Cornelissen I., Amorim R., Han C., Sukumaran S., Janssen L., Suls T. (2022). Characterization of JNJ-80948543, a novel CD79bxCD20xCD3 trispecific T-cell redirecting antibody for the treatment of b-cell non-Hodgkin lymphoma. Blood.

[38] Fontan L., Zwolak A., Guimerans-Lorenzo I., Bekkers M., Hein N., Vloemans N., Trella E., Smets T., Cornelissen I., Assefa A. (2024). JNJ-87801493 (CD20xCD28), a Potential First-in-Class CD20 Targeted CD28 Costimulatory Bispecific Antibody, Enhances the Activity of B-Cell Targeting T-Cell Engagers in Preclinical Models. Blood.

[39] Cao Z., Osellame L.D., Allan L., Scott A.M. (2025). Clinical development of tri-specific antibodies for immune-oncology. Expert Opin. Investig. Drugs.

[40] Horenstein A.L., Faini A.C., Morandi F., Ortolan E., Storti P., Giuliani N., Richardson P.G., Malavasi F. (2025). Monoclonal anti-CD38 therapy in human myeloma: Retrospects and prospects. Front. Immunol..

[41] Kantarjian H., Stein A., Gökbuget N., Fielding A.K., Schuh A.C., Ribera J.M., Wei A., Dombret H., Foà R., Bassan R. (2017). Blinatumomab versus chemotherapy for advanced acute lymphoblastic leukemia. N. Engl. J. Med..

[42] Brown P.A., Ji L., Xu X., Devidas M., Hogan L.E., Borowitz M.J., Raetz E.A., Zugmaier G., Sharon E., Bernhardt M.B. (2021). Effect of postreinduction therapy consolidation with blinatumomab vs. chemotherapy on disease-free survival in children, adolescents, and young adults with first relapse of B-cell acute lymphoblastic leukemia: A randomized clinical trial. JAMA.

[43] Yao Y., Hu Y., Wang F. (2023). Trispecific antibodies for cancer immunotherapy. Immunology.

[44] Huang S., Van Duijnhoven S.M., Sijts A.J., Van Elsas A. (2020). Bispecific antibodies targeting dual tumor-associated antigens in cancer therapy. J. Cancer Res. Clin. Oncol..

[45] Zabaleta A., Blanco L., Kim P., Bisht K., Wang H., Van de Velde H.J., Lasa M., Tamariz-Amador L.E., Otero P.R., San Miguel J. (2023). A CD38/CD28xCD3 trispecific T-cell engager (TCE) as a potentially active agent in multiple myeloma patients relapsed and/or refractory (RRMM) to anti-CD38 monoclonal antibodies (mAbs). Blood.

[46] Müller D. (2022). Optimized CD19/CD22/CD3 antibody. Blood J. Am. Soc. Hematol..

[47] Uy G.L., Aldoss I., Foster M.C., Sayre P.H., Wieduwilt M.J., Advani A.S., Godwin J.E., Arellano M.L., Sweet K.L., Emadi A. (2021). Flotetuzumab as salvage immunotherapy for refractory acute myeloid leukemia. Blood J. Am. Soc. Hematol..

[48] Espinoza-Gutarra M.R., Green S.D., Zeidner J.F., Konig H. (2021). *CD*123-targeted therapy in acute myeloid leukemia. Expert Rev. Hematol..

[49] Lee E., Lee S., Park S., Son Y.G., Yoo J., Koh Y., Shin D.Y., Lim Y., Won J. (2023). Asymmetric anti-CLL-1× CD3 bispecific antibody, ABL602 2+ 1, with attenuated CD3 affinity endows potent antitumor activity but limited cytokine release. J. Immunother. Cancer.

[50] Guarnera L., Bravo-Perez C., Visconte V. (2023). Immunotherapy in acute myeloid leukemia: A literature review of emerging strategies. Bioengineering.

[51] Ravandi F., Bashey A., Stock W., Foran J.M., Mawad R., Egan D., Blum W., Yang A., Pastore A., Johnson C. (2020). Complete responses in relapsed/refractory acute myeloid leukemia (AML) patients on a weekly dosing schedule of vibecotamab (XmAb14045), a CD123 x CD3 T cell-engaging bispecific antibody; initial results of a phase 1 study. Blood.

[52] Sallman D.A., Al Malki M., Asch A.S., Lee D.J., Kambhampati S., Donnellan W.B., Bradley T.J., Vyas P., Jeyakumar D., Marcucci G. (2020). Tolerability and efficacy of the first-in-class anti-CD47 antibody magrolimab combined with azacitidine in MDS and AML patients: Phase Ib results. J. Clin. Oncol..

[53] Leong S.R., Sukumaran S., Hristopoulos M., Totpal K., Stainton S., Lu E., Wong A., Tam L., Newman R., Vuillemenot B.R. (2017). An anti-CD3/anti–CLL-1 bispecific antibody for the treatment of acute myeloid leukemia. Blood J. Am. Soc. Hematol..

[54] Arvindam U.S., van Hauten P.M., Schirm D., Schaap N., Hobo W., Blazar B.R., Vallera D.A., Dolstra H., Felices M., Miller J.S. (2021). A trispecific killer engager molecule against CLEC12A effectively induces NK-cell mediated killing of AML cells. Leukemia.

[55] Rolin C., Zimmer J., Seguin-Devaux C. (2024). Bridging the gap with multispecific immune cell engagers in cancer and infectious diseases. Cell. Mol. Immunol..

[56] Roskopf C.C., Braciak T.A., Fenn N.C., Kobold S., Fey G.H., Hopfner K.P., Oduncu F.S. (2016). Dual-targeting triplebody 33-3-19 mediates selective lysis of biphenotypic CD19+ CD33+ leukemia cells. Oncotarget.

[57] Bannerji R., Allan J.N., Arnason J.E., Brown J.R., Advani R., Ansell S.M., O’Brien S.M., Duell J., Martin P., Joyce R.M. (2020). *Odronextamab (REGN*1979), a human CD20 x CD3 bispecific antibody, induces durable, complete responses in patients with highly refractory B-cell non-Hodgkin lymphoma, including patients refractory to CAR T therapy. Blood.

[58] Hutchings M. (2023). The evolving therapy of DLBCL: Bispecific antibodies. Hematol. Oncol..

[59] Morschhauser F., Fowler N.H., Feugier P., Bouabdallah R., Tilly H., Palomba M.L., Fruchart C., Libby E.N., Casasnovas R.O., Flinn I.W. (2018). Rituximab plus lenalidomide in advanced untreated follicular lymphoma. N. Engl. J. Med..

[60] Budde L.E., Sehn L.H., Matasar M., Schuster S.J., Assouline S., Giri P., Kuruvilla J., Canales M., Dietrich S., Fay K. (2022). Safety and efficacy of mosunetuzumab, a bispecific antibody, in patients with relapsed or refractory follicular lymphoma: A single-arm, multicentre, phase 2 study. Lancet Oncol..

[61] Dickinson M.J., Carlo-Stella C., Morschhauser F., Bachy E., Corradini P., Iacoboni G., Khan C., Wróbel T., Offner F., Trněný M. (2022). Glofitamab for relapsed or refractory diffuse large B-cell lymphoma. N. Engl. J. Med..

[62] Matasar M., Bartlett N.L., Shadman M., Budde L.E., Flinn I., Gregory G.P., Kim W.S., Hess G., El-Sharkawi D., Diefenbach C.S. (2024). Mosunetuzumab safety profile in patients with relapsed/refractory B-cell non-hodgkin lymphoma: Clinical management experience from a pivotal phase I/II trial. Clin. Lymphoma Myeloma Leuk..

[63] Brody J., Falchi L., Vitolo U., Nijland M., Offner F., Snauwaert S., Patah P., Marek J., Morehouse C., Steele A.J. (2024). Fixed-Duration Epcoritamab in Combination with Bendamustine+ Rituximab for First-Line Treatment of Follicular Lymphoma: Initial Results from Epcore NHL-2 Arm 3. Blood.

[64] Budde L.E., Sehn L.H., Matasar M.J., Schuster S.J., Assouline S., Giri P., Kuruvilla J., Canales M., Dietrich S., Fay K. (2021). Mosunetuzumab monotherapy is an effective and well-tolerated treatment option for patients with relapsed/refractory (R/R) follicular lymphoma (FL) who have received ≥ 2 prior lines of therapy: Pivotal results from a phase I/II study. Blood.

[65] Linton K.M., Wahlin B., Leppa S., Morschhauser F., Elliott B., Liu T., Stirner M.C., Abbas A., Falchi L. (2022). Subcutaneous Epcoritamab in Combination with Rituximab and Lenalidomide in Relapsed or Refractory Follicular Lymphoma: Preliminary Phase 1/2 Results. Br. J. Haematol..

[66] Lu H., Oka A., Coulson M., Polli J.R., Aardalen K., Ramones M., Walker D.B., Carrion A., Alexander D., Klopfenstein M. (2022). PIT565, a first-in-class anti-CD19, anti-CD3, anti-CD2 trispecific antibody for the treatment of B cell malignancies. Blood.

[67] Mazza I.A., Barba P., Yuda J., Palomba M.L., Alderuccio J.P., De Vriendt C., Corradini P., Lim F.L., Zinzani P.L., Jain N. (2023). A phase 1 study of PIT565, a first-in-class, anti-CD3, anti-CD19, anti-CD2 trispecific antibody in patients with relapsed and/or refractory B-Cell malignancies. Blood.

[68] Abou Dalle I., Dulery R., Moukalled N., Ricard L., Stocker N., El-Cheikh J., Mohty M., Bazarbachi A. (2024). Bi-and Tri-specific antibodies in non-Hodgkin lymphoma: Current data and perspectives. Blood Cancer J..

[69] Vasu S., Bezerra E., Denlinger N., Szuminski N., Schneider D., Dash P., Wirthlin L., Epperla N., Sawalha Y., Woyach J.A. (2024). Initial Results of a First-in-Human, Phase I Study Point-of-Care Manufacturing of Trispecific CAR-T Cells Targeting CD19/20/22 in B-Cell Malignancies. Blood.

[70] Qureshi Z., Jamil A., Altaf F., Siddique R., Ahmed F. (2024). Efficacy and safety of teclistamab in relapsed or refractory multiple myeloma: A systematic review and meta-analysis. Ann. Hematol..

[71] Tomasson M.H., Iida S., Niesvizky R., Mohty M., Bahlis N.J., Martinez‐Lopez J., Koehne G., Rodriguez-Otero P., Prince H.M., Viqueira A. (2024). Long-term survival and safety of elranatamab in patients with relapsed or refractory multiple myeloma: Update from the MagnetisMM-3 study. HemaSphere.

[72] Lee H.C., Bumma N., Richter J.R., Dhodapkar M.V., Hoffman J.E., Suvannasankha A., Zonder J.A., Shah M.R., Lentzsch S., Maly J.J. (2023). LINKER-MM1 study: Linvoseltamab (REGN5458) in patients with relapsed/refractory multiple myeloma. J. Clin. Oncol..

[73] Chari A., Minnema M.C., Berdeja J.G., Oriol A., van de Donk N.W., Rodríguez-Otero P., Askari E., Mateos M.V., Costa L.J., Caers J. (2022). Talquetamab, a T-cell–redirecting GPRC5D bispecific antibody for multiple myeloma. N. Engl. J. Med..

[74] Trudel S., Cohen A.D., Krishnan A.Y., Fonseca R., Spencer A., Berdeja J.G., Lesokhin A., Forsberg P.A., Laubach J.P., Costa L.J. (2021). Cevostamab monotherapy continues to show clinically meaningful activity and manageable safety in patients with heavily pre-treated relapsed/refractory multiple myeloma (RRMM): Updated results from an ongoing phase I study. Blood.

[75] Yan S., Ming X., Zheng R., Zhu X., Xiao Y. (2025). Application of GPRC5D Targeting Therapy in Relapsed Refractory Multiple Myeloma. Cancer Med..

[76] van de Donk N.W., Vega G., Perrot A., Anguille S., Oriol A., Minnema M., Kaiser M.F., Lee H.C., Garfall A., Matous J.V. First-in-Human Study of JNJ-79635322 (JNJ-5322), a Novel, Next-Generation Trispecific Antibody (TsAb), in Patients (pts) with Relapsed/Refractory Multiple Myeloma (RRMM): Initial Phase 1 Results. https://meetings.asco.org/abstracts-presentations/243590.

[77] Grab A.L., Kim P.S., John L., Bisht K., Wang H., Baumann A., Van de Velde H., Sarkar I., Shome D., Reichert P. (2024). Pre-Clinical Assessment of SAR442257, a CD38/CD3xCD28 Trispecific T Cell Engager in Treatment of Relapsed/Refractory Multiple Myeloma. Cells.

[78] Wu L., Seung E., Xu L., Rao E., Lord D.M., Wei R.R., Cortez-Retamozo V., Ospina B., Posternak V., Ulinski G. (2020). Trispecific antibodies enhance the therapeutic efficacy of tumor-directed T cells through T cell receptor co-stimulation. Nat. Cancer.

[79] van de Donk N.W., Zweegman S. (2023). T-cell-engaging bispecific antibodies in cancer. Lancet.

[80] Gökbuget N., Dombret H., Bonifacio M., Reichle A., Graux C., Faul C., Diedrich H., Topp M.S., Brüggemann M., Horst H.A. (2018). Blinatumomab for minimal residual disease in adults with B-cell precursor acute lymphoblastic leukemia. Blood J. Am. Soc. Hematol..

[81] Sangwan K., Sharma V., Goyal P.K. (2024). Pharmacological profile of novel anti-cancer drugs approved by USFDA in 2022: A review. Curr. Mol. Med..

[82] Schjesvold F., Jelinek T., Polgarova K., Pour L., Yoon S.S., Kim W.S., Fosså A., San-Miguel J.F., Canales M., Rodríguez-Otero P. (2024). First-in-Human Phase 1 Study of SAR442257 in Patients with Relapsed/Refractory Multiple Myeloma and Non-Hodgkin Lymphoma. Blood.

[83] Martin T.G., Mateos M.V., Nooka A., Banerjee A., Kobos R., Pei L., Qi M., Verona R., Doyle M., Smit J. (2023). Detailed overview of incidence and management of cytokine release syndrome observed with teclistamab in the MajesTEC-1 study of patients with relapsed/refractory multiple myeloma. Cancer.

[84] Abramson J.S., Ku M., Hertzberg M., Huang H.Q., Fox C.P., Zhang H., Yoon D.H., Kim W.S., Abdulhaq H., Townsend W. (2024). Glofitamab plus gemcitabine and oxaliplatin (GemOx) versus rituximab-GemOx for relapsed or refractory diffuse large B-cell lymphoma (STARGLO): A global phase 3, randomised, open-label trial. Lancet.

[85] Thieblemont C., Phillips T., Ghesquieres H., Cheah C.Y., Clausen M.R., Cunningham D., Do Y.R., Feldman T., Gasiorowski R., Jurczak W. (2023). Epcoritamab, a novel, subcutaneous CD3xCD20 bispecific T-cell–engaging antibody, in relapsed or refractory large B-cell lymphoma: Dose expansion in a phase I/II trial. J. Clin. Oncol..

[86] Nolan-Stevaux O., Smith R. (2024). Logic-gated and contextual control of immunotherapy for solid tumors: Contrasting multi-specific T cell engagers and CAR-T cell therapies. Front. Immunol..

[87] Locke F.L., Mahmoudjafari Z., Kebriaei P., Gardner R.A., Frigault M.J., Frey N., Komanduri K.V., Perales M.A., Nikiforow S. (2025). Awakening from REMS: ASTCT 80/20 Ongoing Recommendations for Safe Use of Chimeric Antigen Receptor T Cells. Transplant. Cell. Ther..

[88] Boutin L., Barjon C., Lafrance L., Senechal E., Bourges D., Vigne E., Scotet E. (2023). Targeting human γδ T cells as a potent and safe alternative to pan-T cells bispecific cell engagers. bioRxiv.

[89] Radtke K.K., Bender B.C., Li Z., Turner D.C., Roy S., Belousov A., Li C.C. (2025). Clinical Pharmacology of Cytokine Release Syndrome with T-Cell–Engaging Bispecific Antibodies: Current Insights and Drug Development Strategies. Clin. Cancer Res..

[90] Leidy S., Snyder J., Davis J.A., Wesson W., Hess B., Jacobs R., Edmonds M., Ahmed N., Hoffmann M. (2024). Practical Implications of Multi-Institution Cytokine Release Syndrome (CRS) and Immune Effector Cell-Associated Neurotoxicity (ICANS) Rates in Lymphoma Targeted Bispecific Antibodies (BsAb). Blood.

[91] Beltran H., Johnson M.L., Jain P., Schenk E.L., Sanborn R.E., Thompson J.R., Dowlati A., Mamdani H., Aggarwal R.R., Anand B.S. (2024). Updated results from a phase 1/2 study of HPN328, a tri-specific, half-life (T1/2) extended DLL3-targeting T-cell engager in patients (pts) with small cell lung cancer (SCLC) and other neuroendocrine cancers (NEC). J. Clin. Oncol..

[92] van de Donk N.W., Moreau P., Garfall A.L., Bhutani M., Oriol A., Nooka A.K., Martin T.G., Rosiñol L., Mateos M.V., Bahlis N.J. (2023). Long-term follow-up from MajesTEC-1 of teclistamab, a B-cell maturation antigen (BCMA) x CD3 bispecific antibody, in patients with relapsed/refractory multiple myeloma (RRMM). J. Clin. Oncol..

[93] Mohan M., Monge J., Shah N., Luan D., Forsberg M., Bhatlapenumarthi V., Balev M., Patwari A., Cheruvalath H., Bhutani D. (2024). Teclistamab in relapsed refractory multiple myeloma: Multi-institutional real-world study. Blood Cancer J..

[94] Dickinson M.J., Carlo-Stella C., Morschhauser F., Bachy E., Cartron G., Corradini P., Bartlett N.L., Iacoboni G., Khan C., Hertzberg M.S. (2024). Fixed-duration Glofitamab Monotherapy Continues to Demonstrate Durable Responses in Patients with Relapsed or Refractory Large B-Cell Lymphoma: 3-year Follow-Up From a Pivotal Phase II Study. Blood.

[95] Paul S., Jabbour E., Nichols E.D., Short N.J., Kantarjian H. (2025). Blinatumomab for the treatment of acute lymphoblastic leukemia in a real-world setting: Clinical vignettes. Leuk. Lymphoma.

[96] Gao W., Yu J., Sun Y., Song Z., Liu X., Han X., Li L., Qiu L., Zhou S., Qian Z. (2025). Adverse events in the nervous system associated with blinatumomab: A real-world study. BMC Med..

[97] Liu L., Krishnan A. (2023). Talquetamab in multiple myeloma. Haematologica.

[98] Tapia-Galisteo A., Álvarez-Vallina L., Sanz L. (2023). Bi-and trispecific immune cell engagers for immunotherapy of hematological malignancies. J. Hematol. Oncol..

[99] Lee D.W., Santomasso B.D., Locke F.L., Ghobadi A., Turtle C.J., Brudno J.N., Maus M.V., Park J.H., Mead E., Pavletic S. (2019). ASTCT consensus grading for cytokine release syndrome and neurologic toxicity associated with immune effector cells. Biol. Blood Marrow Transplant..

[100] Carrara S.C. (2023). Generation of Multispecific Antibodies with Immune Cell Modulating Functions. Ph.D. Thesis.

[101] Li H., Zhao L., Sun Z., Yao Y., Li L., Wang J., Hua T., Ji S., Wang S., Cheng H. (2022). Prolonged hematological toxicity in patients receiving BCMA/CD19 CAR-T-cell therapy for relapsed or refractory multiple myeloma. Front. Immunol..

[102] Moreau P., Garfall A.L., van de Donk N.W., Nahi H., San-Miguel J.F., Oriol A., Nooka A.K., Martin T., Rosinol L., Chari A. (2022). Teclistamab in relapsed or refractory multiple myeloma. N. Engl. J. Med..

[103] Dima D., Davis J.A., Ahmed N., Sannareddy A., Shaikh H., Mahmoudjafari Z., Khouri J., Kaur G., Strouse C., Valent J. (2023). Real-world safety and efficacy of teclistamab for patients with heavily pretreated relapsed-refractory multiple myeloma. Blood.

[104] Huang Y., Zhang X., Zhang R., Jing Z., Zhao C., Pan F., Zheng B., Dai R., Yang Y., Zeng L. (2025). Beyond antibodies and CAR-T: CC312, a first-in-class, anti-CD19, anti-CD3, anti-CD28 trispecific antibody in treatment with relapsed and/or refractory B-cell malignancies. Cancer Res..

[105] Menon V., Holkova B., Pacaud L., Gn S., Garton A., Pihlgren M., Matsuura T., van der Graaf P.H., Perro M., Konto C. (2025). Clinical validation of a quantitative systems pharmacology (QSP) model of ISB 2001 used for deriving first in human (FIH) dose and efficient phase 1 dose escalation design in relapsed refractory multiple myeloma (RRMM) patients. Cancer Res..

[106] Tan Y., Li X., Yu F., Xu J., Qian Z., Cao Y., Yang X., Du Q., Peng F., Han S. (2024). Abstract LB128: A novel tri-specific T cell engager targeting BCMA and GPRC5D for treatment of multiple myeloma. Cancer Res..

[107] Roth H., Rogers D., Sanchez I., Tyrell B., Snyder A., Doolan K., Doranz B., Chambers R., Rucker J. (2025). GPRC5D multispecific antibodies with potent anti-tumor activity against multiple myeloma. Cancer Res..

[108] Bangolo A., Amoozgar B., Mansour C., Zhang L., Gill S., Ip A., Cho C. (2025). Comprehensive Review of Early and Late Toxicities in CAR T-Cell Therapy and Bispecific Antibody Treatments for Hematologic Malignancies. Cancers.

[109] Salvaris R., Ong J., Gregory G.P. (2021). Bispecific antibodies: A review of development, clinical efficacy and toxicity in B-cell lymphomas. J. Pers. Med..

[110] Tan C.R., Asoori S., Huang C.Y., Brunaldi L., Popat R., Kastritis E., Martinez-Lopez J., Bansal R., Silva Corraes A.D., Chhabra S. (2025). Real-world evaluation of teclistamab for the treatment of relapsed/refractory multiple myeloma (RRMM): An International Myeloma Working Group Study. Blood Cancer J..

[111] Hutchings M., Morschhauser F., Iacoboni G., Carlo-Stella C., Offner F.C., Sureda A., Salles G., Martínez-Lopez J., Crump M., Thomas D.N. (2021). Glofitamab, a novel, bivalent CD20-targeting T-cell–engaging bispecific antibody, induces durable complete remissions in relapsed or refractory B-cell lymphoma: A phase I trial. J. Clin. Oncol..

[112] Elemian S., Habbas A., Jumean S., Al Omour B., Hamad M., Tan J.Y., Chan K.H., Guron G., Shaaban H. (2024). Efficacy and Safety of Mosunetuzumab in Relapsed/Refractory Non-Hodgkin Lymphoma: A Systematic Review. Blood.

[113] An G., Xing L., Chen W., Zhang Y., Gao W., Qiu L.G., Wu G., Ning J., Wei M., Li F. (2024). MBS314, a G Protein-Coupled Receptor Family C Group 5 Member D (GPRC5D) x B-Cell Maturation Antigen (BCMA) x CD3 Trispecific Antibody, in Relapsed and/or Refractory Multiple Myeloma (RRMM): Preliminary Results from a Phase I, First-in-Human, Open-Label, Dose Escalation Study. Blood.

[114] Mazahreh F., Mazahreh L., Schinke C., Thanendrarajan S., Zangari M., Shaughnessy J.D., Zhan F., Van Rhee F., Al Hadidi S. (2023). Risk of infections associated with the use of bispecific antibodies in multiple myeloma: A pooled analysis. Blood Adv..

[115] Martino M., Gamberi B., Antonioli E., Aquino S., Della Pepa R., Malerba L., Mangiacavalli S., Pezzatti S., Bringhen S., Zamagni E. (2024). Anti-BCMA CAR-T cell-based therapies and bispecific antibodies in the immunotherapy era: Are we ready for this?. Expert Rev. Hematol..

[116] Yee A.J. (2024). Improving outcomes with anti-BCMA bispecific antibodies with attention to infection. Blood Cancer J..

[117] Rafei H., Rezvani K. (2024). Mitigating infection risks: The promise and challenge of bispecific antibodies in haematological malignancies. Br. J. Haematol..

[118] Fu B., Liu R., Gao G., Lin Z., He A. (2024). Mechanisms and salvage treatments in patients with multiple myeloma relapsed post-BCMA CAR-T cell therapy. Front. Immunol..

[119] Lee H., Ahn S., Maity R., Leblay N., Ziccheddu B., Truger M., Chojnacka M., Cirrincione A., Durante M., Tilmont R. (2023). Mechanisms of antigen escape from BCMA-or GPRC5D-targeted immunotherapies in multiple myeloma. Nat. Med..

[120] Woo S.R., Turnis M.E., Goldberg M.V., Bankoti J., Selby M., Nirschl C.J., Bettini M.L., Gravano D.M., Vogel P., Liu C.L. (2012). Immune inhibitory molecules LAG-3 and PD-1 synergistically regulate T-cell function to promote tumoral immune escape. Cancer Res..

[121] Ghermezi M., Li M., Vardanyan S., Harutyunyan N.M., Gottlieb J., Berenson A., Spektor T.M., Andreu-Vieyra C., Petraki S., Sanchez E. (2016). Serum B-cell maturation antigen: A novel biomarker to predict outcomes for multiple myeloma patients. Haematologica.

[122] Del Giudice M.L., Galimberti S., Buda G. (2023). Beyond BCMA, why GPRC5D could be the right way: Treatment strategies with immunotherapy at relapse after anti-BCMA agents. Cancer Immunol. Immunother..

[123] Fernández de Larrea C., Staehr M., Lopez A.V., Ng K.Y., Chen Y., Godfrey W.D., Purdon T.J., Ponomarev V., Wendel H.G., Brentjens R.J. (2020). Defining an optimal dual-targeted CAR T-cell therapy approach simultaneously targeting BCMA and GPRC5D to prevent BCMA escape–driven relapse in multiple myeloma. Blood Cancer Discov..

[124] Lee H., Neri P., Ahn S., Maity R., Leblay N., Ziccheddu B., Chojnacka M., Tilmont R., Barakat E., Landgren O. (2022). Role of TNFRSF17 and GPRC5D structural and point mutations in resistance to targeted immunotherapies in multiple myeloma (MM). Blood.

[125] Hofmann M., Thimme R., Schamel W.W. (2024). PD-1 and LAG-3: Synergistic fostering of T cell exhaustion. Signal Transduct. Target. Ther..

[126] Lichtenegger F.S., Rothe M., Schnorfeil F.M., Deiser K., Krupka C., Augsberger C., Schlüter M., Neitz J., Subklewe M. (2018). Targeting LAG-3 and PD-1 to enhance T cell activation by antigen-presenting cells. Front. Immunol..

[127] Munshi N.C., Avet-Loiseau H., Rawstron A.C., Owen R.G., Child J.A., Thakurta A., Sherrington P., Samur M.K., Georgieva A., Anderson K.C. (2017). Association of minimal residual disease with superior survival outcomes in patients with multiple myeloma: A meta-analysis. JAMA Oncol..

[128] Garfall A.L., Nooka A.K., van de Donk N.W., Moreau P., Bhutani M., Oriol A., Martin T.G., Rosiñol L., Mateos M.V., Bahlis N. (2024). *MM*-336 Long-Term Follow-Up from the Phase 1/2 MajesTEC-1 Trial of Teclistamab in Patients With Relapsed/Refractory Multiple Myeloma (RRMM). Clin. Lymphoma Myeloma Leuk..

[129] Lesokhin A.M., Tomasson M.H., Arnulf B., Bahlis N.J., Miles Prince H., Niesvizky R., Rodrίguez-Otero P., Martinez-Lopez J., Koehne G., Touzeau C. (2023). Elranatamab in relapsed or refractory multiple myeloma: Phase 2 MagnetisMM-3 trial results. Nat. Med..

[130] Avet-Loiseau H., Ludwig H., Landgren O., Paiva B., Morris C., Yang H., Zhou K., Ro S., Mateos M.V. (2020). Minimal residual disease status as a surrogate endpoint for progression-free survival in newly diagnosed multiple myeloma studies: A meta-analysis. Clin. Lymphoma Myeloma Leuk..

[131] Perrot A., Lauwers-Cances V., Corre J., Robillard N., Hulin C., Chretien M.L., Dejoie T., Maheo S., Stoppa A.M., Pegourie B. (2018). Minimal residual disease negativity using deep sequencing is a major prognostic factor in multiple myeloma. Blood J. Am. Soc. Hematol..

[132] Xia J., Li Z., Xu K. (2023). Immunotherapies targeting GPRC5D in relapsed or refractory multiple myeloma: Latest updates from 2022 ASH Annual Meeting. J. Hematol. Oncol..

[133] Falchi L., Carlo-Stella C., Morschhauser F., Hutchings M., Bachy E., Cartron G., Khan C., Tani M., Martinez-Lopez J., Bartlett N.L. (2023). Glofitamab monotherapy in pts with relapsed/refractory (R/R) large B-cell lymphoma (LBCL): Extended follow-up and landmark analyses from a pivotal phase II study. J. Clin. Oncol..

[134] Balendran S., Tam C., Ku M. (2023). T-Cell Engaging Antibodies in Diffuse Large B Cell Lymphoma—An Update. J. Clin. Med..

[135] Zinselmeyer B.H., Heydari S., Sacristán C., Nayak D., Cammer M., Herz J., Cheng X., Davis S.J., Dustin M.L., McGavern D.B. (2013). PD-1 promotes immune exhaustion by inducing antiviral T cell motility paralysis. J. Exp. Med..

[136] Paiva B., Gaffney B., Burnett K., Castiglioni P., Angelo M., Pierce D.W., Boss I.W. (2022). Synergistic antitumor activity of alnuctamab (ALNUC.; BMS-986349; CC-93269), a BCMA 2+ 1 T cell engager (TCE), and celmod agents in multiple myeloma (MM) preclinical models. Blood.

[137] Haber L., Olson K., Kelly M.P., Crawford A., DiLillo D.J., Tavaré R., Ullman E., Mao S., Canova L., Sineshchekova O. (2021). Generation of T-cell-redirecting bispecific antibodies with differentiated profiles of cytokine release and biodistribution by CD3 affinity tuning. Sci. Rep..

[138] Labanca C., Martino E.A., Vigna E., Bruzzese A., Mendicino F., De Luca P., Lucia E., Olivito V., Fragliasso V., Neri A. (2024). Mosunetuzumab for the treatment of follicular lymphoma. Expert Opin. Biol. Ther..

[139] Sun L., Romancik J.T. (2025). The Development and Application of Bispecific Antibodies in B-Cell Non-Hodgkin Lymphoma. J. Pers. Med..

[140] Bruzzese A., Martino E.A., Labanca C., Caridà G., Mendicino F., Lucia E., Olivito V., Puccio N., Neri A., Morabito F. (2025). Therapeutic Strategies for Relapsed or Refractory B-Cell Acute Lymphoblastic Leukemia in Adult Patients: Optimizing the Use of Monoclonal Antibodies. Eur. J. Haematol..

[141] Peter J., Toppeta F., Trubert A., Danhof S., Hudecek M., Däullary T. (2025). Multi-Targeting CAR-T Cell Strategies to Overcome Immune Evasion in Lymphoid and Myeloid Malignancies. Oncol. Res. Treat..

[142] Cliff E.R., Mian H., Mohyuddin G.R. (2022). Teclistamab in relapsed or refractory multiple myeloma. N. Engl. J. Med..

[143] Shaver J., Horton D., Halford Z. (2025). Targeting GPRC5D With Talquetamab: A New Frontier in Bispecific Antibody Therapy for Relapsed/Refractory Multiple Myeloma. Ann. Pharmacother..

[144] Xu Y., Cai Z., Xia Z., Yang C., Chen J., Zhu Z., Jing X., Tian J., Zhang N., Cui A. (2024). A Phase I First-in-Human, Open-Label Trial to Investigate the Safety, Tolerability, Pharmacokinetics and Preliminary Antitumor Activity of SIM0500, a Humanized GPRC5D-BCMA-CD3 Trispecific Antibody, in Participants with Relapsed or Refractory Multiple Myeloma. Blood.

[145] Carrara S.C., Harwardt J., Grzeschik J., Hock B., Kolmar H. (2022). TriTECM: A tetrafunctional T-cell engaging antibody with built-in risk mitigation of cytokine release syndrome. Front. Immunol..

[146] Zhang T., Lin Y., Gao Q. (2023). Bispecific antibodies targeting immunomodulatory checkpoints for cancer therapy. Cancer Biol. Med..

[147] Desnoyers L.R., Vasiljeva O., Richardson J.H., Yang A., Menendez E.E., Liang T.W., Wong C., Bessette P.H., Kamath K., Moore S.J. (2013). Tumor-specific activation of an EGFR-targeting probody enhances therapeutic index. Sci. Transl. Med..

[148] Grymula K., Tarnowski M., Wysoczynski M., Drukala J., Barr F.G., Ratajczak J., Kucia M., Ratajczak M.Z. (2010). Overlapping and distinct role of CXCR7-SDF-1/ITAC and CXCR4-SDF-1 axes in regulating metastatic behavior of human rhabdomyosarcomas. Int. J. Cancer.

[149] FDAU (2024). FDA Grants Accelerated Approval to Tarlatamab-Dlle for Extensive Stage Small Cell Lung Cancer. https://www.lungcancerstoday.com/post/fda-grants-accelerated-approval-to-tarlatamab-dlle-for-extensive-stage-sclc.

[150] Hummel H.D., Kufer P., Grüllich C., Seggewiss-Bernhardt R., Deschler-Baier B., Chatterjee M., Goebeler M.E., Miller K., de Santis M., Loidl W. (2021). Pasotuxizumab, a BiTE® immune therapy for castration-resistant prostate cancer: Phase I, dose-escalation study findings. Immunotherapy.

[151] Gedeon P.C., Schaller T.H., Chitneni S.K., Choi B.D., Kuan C.T., Suryadevara C.M., Snyder D.J., Schmittling R.J., Szafranski S.E., Cui X. (2018). A rationally designed fully human EGFRvIII: CD3-targeted bispecific antibody redirects human T cells to treat patient-derived intracerebral malignant glioma. Clin. Cancer Res..

[152] Leitao C.D., Borras A.M., Xu T., Oroujeni M., Liu Y., Westerberg C., Clinton J., Tolmachev V., Orlova A., Ståhl S. (2023). Conditionally activated affibody-based prodrug targeting EGFR demonstrates improved tumour selectivity. J. Control. Release.

[153] Li D., Cheng P., Wang J., Qiu X., Zhang X., Xu L., Liu Y., Qin S. (2019). IRF6 is directly regulated by ZEB1 and ELF3, and predicts a favorable prognosis in gastric cancer. Front. Oncol..

[154] Marinov T.M., Wasdin P.T., Jordaan G., Janke A.K., Abu-Shmais A.A., Georgiev I.S. (2025). An expandable synthetic library of human paired antibody sequences. PLOS Comput. Biol..

[155] Prelaj A., Galli E.G., Miskovic V., Pesenti M., Viscardi G., Pedica B., Mazzeo L., Bottiglieri A., Provenzano L., Spagnoletti A. (2023). Real-world data to build explainable trustworthy artificial intelligence models for prediction of immunotherapy efficacy in NSCLC patients. Front. Oncol..

[156] Tran K.A., Addala V., Koufariotis L.T., Zhang J., Wood S., Leonard C., Hoeijmakers L.L., Blank C.U., Crispin-Ortuzar M., Williams E.D. (2025). Explainable machine learning identifies features and thresholds predictive of immunotherapy response. bioRxiv.

[157] BioCopy (2024). BioCopy Chooses Genedata for AI-Powered Multispecific Antibody Development. https://www.genedata.com/company/news/details/press-release/biocopy-advances-ai-driven-antibody-discovery.

